# Whole-genome sequence of the type strain and two field strains of *Arsenicicoccus dermatophilus* causing pododermatitis in greater flamingos (*Phoenicopterus roseus*)

**DOI:** 10.1128/mra.00704-26

**Published:** 2026-09-17

**Authors:** Alexandra Rossano, Stefanie Gobeli Brawand, Javier E. Fernandez, Andreas Thomann, Vincent Perreten

**Affiliations:** 1 Division of Molecular Bacterial Epidemiology and Infectious Diseases, Institute of Veterinary Bacteriology, Vetsuisse Faculty, University of Bern 27210, Bern, Switzerland; 2 Federal Food Safety and Veterinary Office FSVO, Bern, Switzerland; 3 Institute of Veterinary Bacteriology, Vetsuisse Faculty, University of Bern 27210, Bern, Switzerland; Wellesley College, Wellesley, Massachusetts, USA

**Keywords:** dermatitis, veterinary pathogens

## Abstract

The complete genome sequence of the type strain KM894/11^T^ and two field strains of *Arsenicicoccus dermatophilus* (KM18/12; KM9/12) isolated from foot skin lesions of captive greater flamingos was determined using Oxford Nanopore and Illumina sequencing technologies. The genomes differ structurally by a large ~650 kb inverted chromosomal fragment and plasmid content.

## ANNOUNCEMENT

*Arsenicicoccus dermatophilus* was first reported in 2013 as the causative agent of pododermatitis in greater flamingos (*Phoenicopterus roseus*), especially affecting birds kept in captivity (1). At the time of its description, identification was only possible through 16S rDNA analysis, DNA-DNA hybridization, and phenotypic observations such as hypha formation (1). No genomes have been reported so far, except for a draft genome of the type strain DSM 25571^T^ (GenBank acc. no. NZ_JAKZHU000000000) (2). Here, we present the complete circular genome of three *A. dermatophilus* strains originally reported (1), including type strain KM894/11^T^ (=DSM 25571^T^) and strains KM18/12 and KM9/12.

The strains were isolated from curetted foot skin lesions of three captive greater flamingos in Switzerland (Table 1) (1). Samples were suspended in 100 µL 0.85% NaCl, and a loopful was streaked on tryptone soy agar containing 5% sheep blood (TSA-SB; Becton Dickinson) and 1,000 IU polymyxin B/mL. Plates were incubated at 37°C for 48 h, and colonies whose cells formed hyphae were selected (1). The strains were cryopreserved in our collection, and KM894/11^T^ was deposited in national collections under respective names: DSM 25571^T^ (German Collection of Microorganisms and Cell Cultures GmbH), CCUG 62181^T^ (Culture Collection University of Gothenburg), and CCOS 690^T^ (Culture Collection of Switzerland). Genomic DNA was extracted from the lawn of colonies grown on TSA-SB at 37°C for 48 h using the Promega Wizard HMW DNA Extraction Kit and purified with CleanNGS magnetic beads (CleanNA). DNA was sequenced using a NEBNext Ultra II Directional DNA Library with TruSeq Adapters on an Illumina NovaSeq 6000 system (2 × 150 bp paired-end, S4 flow cell, 300 cycles; NGS Platform, University of Bern), and with the MinION MK1b device on an R9.4.1 Flongle flow cell using the 1D ligation kit (SQK-LSK109; Oxford Nanopore Technology, ONT). ONT reads were basecalled using Dorado v7.2.13 (3) and quality-controlled using NanoPlot v1.40.2 (4). Illumina reads were quality-controlled by FastQC v0.11.9 (5), quality-filtered, and trimmed using Trimmomatic v0.4 (6). ONT reads were *de novo* assembled using Trycycler v0.5.4 (7). The resulting circular assemblies were polished with paired-end Illumina reads using Polypolish v0.6.0 (8) and annotated by NCBI PGAP v6.5/v6.9 upon GenBank submission (9). Chromosomes were rotated to DnaA using Geneious Prime v2022.2.2 (Biomatters Ltd.), whereas plasmids remained unrotated. Single nucleotide polymorphisms (SNPs) were called using Snippy v3.2 (https://github.com/tseemann/snippy). Average nucleotide identity (ANI) was calculated using ANI Calculator (10). Genome alignments were performed using progressive Mauve v1.1.3 (11) and clinker v0.0.26 (12). Default parameters were used for all software. WGS characteristics are listed in Table 1.

**TABLE 1:** Sequencing statistics and genomic characteristics of *Arsenicicoccus dermatophilus* strains KM894/11^T^, KM18/12, and KM9/12 isolated from foot skin lesions of greater flamingos^*c*^

*Arsenicicoccus dermatophilus* strains			
Parameters^*a*^	KM894/11^T^	KM18/12	KM9/12
Origin	Greater flamingo	Greater flamingo	Greater flamingo
Sample	Foot skin lesion	Foot skin lesion	Foot skin lesion
Date of isolation	08/2011	08/2012	08/2012
Place of isolation	IVB, Bern, Switzerland (46°57'25.9"N 7°25'37.3"E)	IVB, Bern, Switzerland (46°57'25.9"N 7°25'37.3"E)	IVB, Bern, Switzerland (46°57'25.9"N 7°25'37.3"E)
Illumina statistics			
SRA accession numbers	SRR24390656	SRR31647406	SRR31647407
Read length (bp)	36–151	36–151	36–151
Number of paired reads	623,537	4,825,868	4,623,033
Coverage (×)	53	441	418
Oxford Nanopore (ONT) statistics			
SRA accession numbers	SRR24390657	SRR31647404	SRR31647405
N50 (bp)	6,836	11,862	10,618
Mean read length (bp)	4,546	8,624	9,447
Number of reads	37,128	73,424	52,593
Coverage (×)	50	193	150
ONT + Illumina Total coverage (×)	103	634	568
Genome			
Chromosome size; acc. no.	3,336,347; CP124757	3,280,977; CP176517	3,311,136; CP176520
Plasmids (size; acc. no.)	pVPAR1 (61,203; CP124758)	pARKM18-1 (61,190; CP176516)	pARKM9-1 (41,638; CP176519)
	pVPAR2 (40,185; CP124759)	pARKM18-2 (53,362; CP176515)	pARKM9-2 (40,533; CP176518)
	pVPAR3 (16,946; CP124760)	pARKM18-3 (40,172; CP176514)	
GC content (%)	72.7	72.7	72.9
Predicted genes	3,146	3,149	3,109
CDSs	3,087	3,090	3,051
Coding genes and CDSs with proteins	3,032	3,042	3,018
rRNAs	2, 2, and 2 (5S, 16S, and 23S)	2, 2, and 2 (5S, 16S, and 23S)	2, 2, 2 (5S, 16S, and 23S)
tRNAs	50	50	49
ncRNAs	3	3	3
pseudogenes	55	48	33
SNPs^*b*^			
KM894/11^T^	–	419	134,359
KM18/12	419	–	134,118
KM9/12	134,359	134,118	–
ANI (%)			
KM894/11^T^	100	99.93	92.21
KM18/12	99.93	100	92.26

^*a*^ CDSs, coding DNA sequences; rRNAs, ribosomal RNAs; tRNAs, transfer RNAs; ncRNAs, non-coding RNAs obtained from PGAP annotations v6.5 for KM894/11^T^ and v6.9 for KM18/12 and KM9/12 upon GenBank submission (9); SNPs, single nucleotide polymorphisms; ANI, average nucleotide identity; acc. no., accession number; IVB, Institute of Veterinary Bacteriology, Bern.

^*b*^ “–” indicates not applicable since it consists of the same strain.

^*c*^ T, type strain.

**Fig 1:**
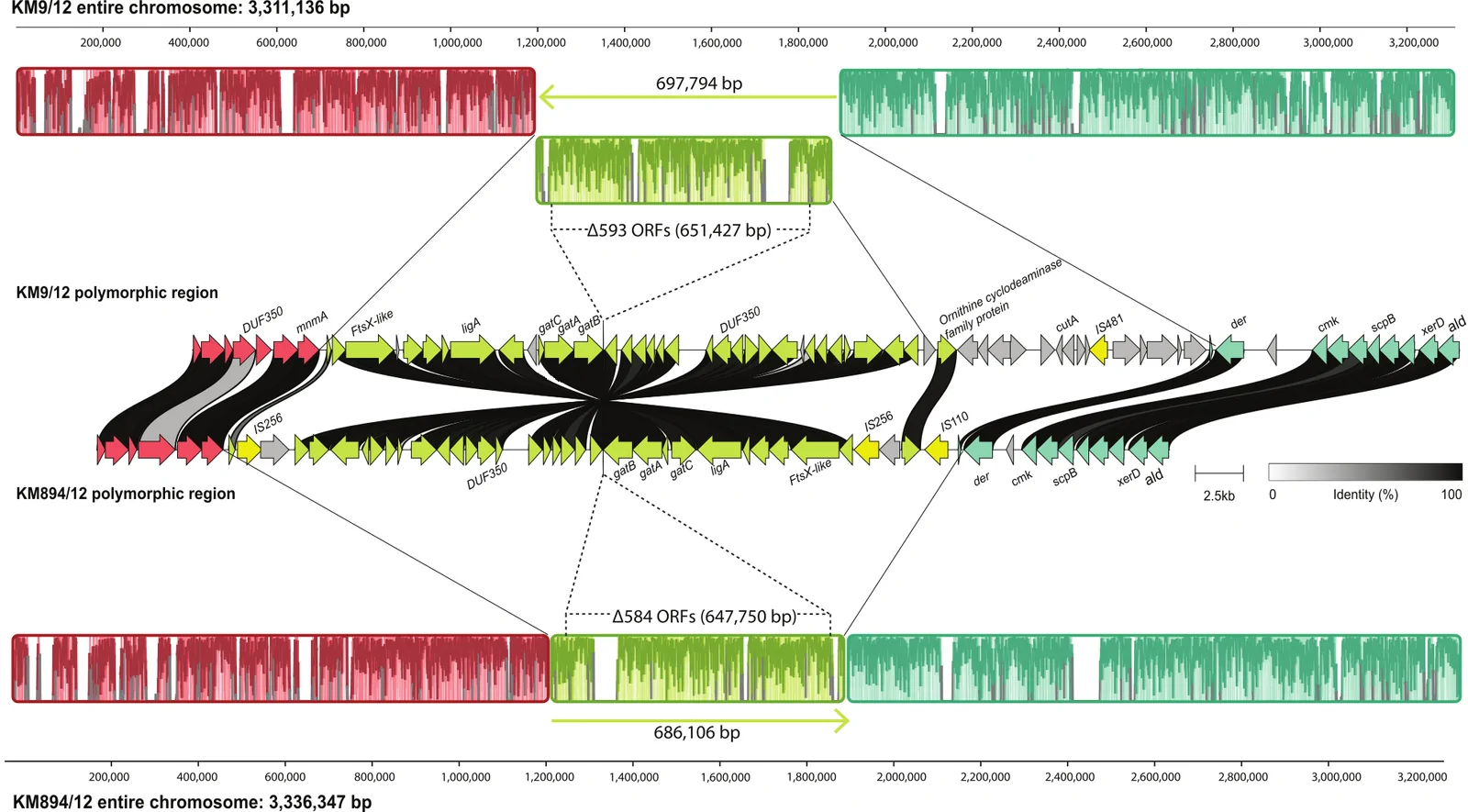
Genome alignments of the complete chromosome of *A. dermatophilus* strains KM894/11^T^ and KM9/12 using the progressive Mauve software v1.1.3, and alignment of the joining regions of the 650-kb polymorphic chromosomal inversion using clinker v0.0.26 and the PGAP-annotated sequences. The conserved genome structures are depicted in red and turquoise blue boxes, and the inverted fragment is in green. Open reading frames (ORFs) are shown for the end and beginning of the inverted fragments and flanking regions and are illustrated by thick arrows. Insertion sequences (IS) are indicated by thick yellow arrows. The rightward thin green arrow indicates the 686,106 bp fragment embedded between *der* and *mnmA* in KM894/11^T^ (identical in KM18/12), and the leftward thin green arrow indicates the corresponding inverted 697,794 bp fragment in KM9/12. The central portion of the inverted fragments (651,427 bp containing 593 ORFs [Δ593 ORFs] in KM9/12 and 647,750 bp containing 584 ORFs [Δ584 ORFs] in KM894/11^T^) was removed from the clinker alignment to obtain a scale that fits within the figure. The final illustration was created using Adobe Illustrator v29.5.1.

The three genomes differed by 419–134,359 SNPs, by the presence of two to three plasmids (Table 1), and by a polymorphic inversion of a large and structurally related chromosomal fragment embedded between core genes *mnmA* and *der* in KM9/12 (697,794 bp) as compared to KM894/11^T^ and KM18/12 (686,106 bp), and likely driven by IS elements (Fig. 1).

These assemblies represent the first fully resolved circular genomes of *A. dermatophilus* and may serve as a baseline for characterizing virulence mechanisms, molecular epidemiology studies, and the development of DNA-based tools for early detection of this pododermatitis pathogen.

## Data Availability

The genome sequence of the three *A. dermatophilus* strains has been deposited in the NCBI database under BioProject accession numbers PRJNA965927 (KM894/11^T^), PRJNA1195889 (KM9/12; KM18/12), BioSample accession numbers SAMN34509338 (KM894/11^T^), SAMN45230874 (KM9/12), and SAMN45230875 (KM18/12), and under GenBank accession numbers CP124757 (chromosome), CP124758 (pVPAR1), CP124759 (pVPAR2), and CP124760 (pVPAR3) (KM894/11^T^); CP176520 (chromosome), CP176519 (pARKM9-1), and CP176518 (pARKM9-2) (KM9/12); CP176517 (chromosome), CP176516 (pARKM18-1), CP176515 (pARKM18-2), and CP176514 (pARKM18-3) (KM18/12). The raw reads were deposited in the SRA database under accession numbers SRS23474794 (SRX27010487 [Illumina], SRX27010489 [ONT]) for KM9/12, (SRS23474795; SRX27010488 [Illumina], SRX27010490 [ONT]) for KM18/12, and SRS17512045; SRX20178795 (Illumina) and SRX20178794 (ONT) for KM894/11^T^.
